# Retrospective single-center study on elderly patients with glioblastoma between 2014 and 2018 evaluating the effect of age and performance status on survival

**DOI:** 10.1093/nop/npac008

**Published:** 2022-01-27

**Authors:** Juha-Matti Pirkkalainen, Anna-Stina Jääskeläinen, Päivi Halonen

**Affiliations:** Faculty of Medicine, University of Helsinki, Helsinki, Finland; Comprehensive Cancer Centre, Helsinki University Hospital, Helsinki, Finland; Comprehensive Cancer Centre, Helsinki University Hospital, Helsinki, Finland

**Keywords:** elderly, glioblastoma, performance status

## Abstract

**Background:**

Incidence of glioblastoma (GBM) increases with age and the prognosis is worse among the elderly. This was shown in a Finnish population-based register study evaluating GBM patients diagnosed between 2000 and 2013. The median overall survival (OS) was poor among the elderly (4.5 months), despite the OS increase during that period. We conducted a study to see if the OS has increased in our hospital area with current therapies.

**Methods:**

One hundred and ninety-eight patients over 65 years at the time of diagnosis, with malignant glioma diagnosed January 1, 2014 to December 31, 2018 at the Helsinki Comprehensive Cancer Center were included. In addition to grade IV gliomas, grade III gliomas with nonmutated R132HIDH1 and only radiographically diagnosed gliomas were included. The demographics and treatment data were collected with performance status evaluated retrospectively. The Kaplan–Meier method and the multivariate Cox proportional hazard model were used for the statistical analysis.

**Results:**

One hundred and seventy-seven patients with grade IV glioma, 6 with grade III glioma with nonmutated IDH1 and 15 radiologically diagnosed patients were included. One hundred and sixteen patients received chemoradiation, 59 only radiotherapy, 3 only temozolomide, and 27 patients did not receive oncological treatments. In the age group 65–70 years the OS was 9.95 months, 70–75 years 10.12 months, and >75 years 5.54 months. Lower WHO status correlated with longer survival independently of the age of the patient. Also methylated O(6)-methylguanine-DNA-methyltransferase and tumor resection correlated with better survival.

**Conclusions:**

The performance status of elderly patients is the most important prognostic factor. When choosing treatment protocols for patients in this age group, the performance status not calendar age should be considered.

Glioblastoma (GBM) has very poor prognosis. Even with the best current standard of care, consisting of tumor resection followed by chemoradiotherapy and adjuvant chemotherapy with temozolomide, patients with GBM that are younger than 70 years reach a median survival of approximately 14.6 months.^1^ According to population-based studies, survival decreases with increasing age.^2,3^ In a report by Ostrom et al.,^4^ the probability of surviving 12 months was only 9.2% for patients 75 years or over compared to 40.7% for patients between 55 and 64 years.

The proportion of elderly patients has been underrepresented in most randomized trials in which the median age has been in average 55 years, whereas the population-based median age for patients with GBM is 65 years.^3^ In 2012, The Nordic randomized phase 3 trial^5^ revealed that for elderly the standard 6-week radiotherapy was associated with poor outcomes in comparison with hypofractionated radiotherapy and temozolomide treatment alone. In their study, the MGMT promoter methylation of the tumor appeared to be a predictive marker for successful treatment with temozolomide only. During the same year, the NOA-08 randomized phase 3 trial^6^ showed noninferiority of temozolomide alone compared with the standard 6-week radiotherapy for elderly patients with GBM.

Perry et al. showed in their randomized prospective study^7^ that addition of temozolomide to hypofractionated radiotherapy in patients 65 years or older with GBM resulted in longer survival than short-course radiotherapy alone (9.3 vs 7.6 months). Korja et al. reported recently in a Finnish retrospective population-based study^8^ that for GBM patients 70 years or over the median survival time increased from 3.6 months in 2000–2006 to 4.5 months in 2007–2013. Even if the median survival increased during the latter period, it is still alarmingly poor and below the international results. However, our clinical experience during the last years regarding the treatment results of the elderly patients with GBM has been more promising than should be expected based on the study of Korja et al.^8^ To study this, we conducted this retrospective single-center study in the Comprehensive Cancer Center at Helsinki University Hospital in Finland. The aim of the study was to find out the real-life outcome and median survival time of elderly patients with GBM treated with current standard therapies during 2014–2018.

## Methods

We identified 198 elderly patients with primary GBM diagnosed between January 1, 2014 and December 31, 2018 at the Helsinki Comprehensive Care Center (CCC). The patients were followed up until October 1, 2020. Here the term elderly refers to patients aged 65 years or older at the time of diagnosis. In addition to patients with grade IV glioma, we included grade III gliomas with nonmutated R132H IDH1 immunohistochemistry as well as radiologically diagnosed GBMs. The radiological diagnosis was always confirmed by a multidisciplinary team assessment.

Demographics and treatment data for the patients were collected retrospectively from the medical records of CCC at Helsinki University Hospital (HUH). This record included all the patients diagnosed with brain tumors either by histology or radiology at the District of Helsinki and Uusimaa. The study was conducted according to the declaration of Helsinki and the International Conference on Harmonization on Good Medical Practices after approval by the Research Unit of CCC at HUH acting according to the trial laws. WHO performance status was retrospectively assessed by an experienced oncologist (A.-S.J.) based on the medical records in which the patient status was described by a clinician responsible for assessing required oncological treatments. The assessment by clinician was carried out postoperatively if surgery or biopsy was performed and before any oncological treatments were given. The performance status was assessed using the Eastern Cooperative Oncology Group performance score.

The statistical analysis was performed using IBM SPSS Statistics 22 for Windows (SPSS, Inc., Chicago, IL) and R Statistical Software (version 4.0.3, R Foundation for Statistical Computing, Vienna, Austria). The Kaplan–Meier method was used to extract overall survival (OS) and progression-free survival (PFS) with 95% confidence intervals. To assess the correlation between OS and potentially relevant covariates, the multivariate Cox proportional hazard model was fitted to the data.

## Results

The patient characteristics are presented in Table 1. One hundred and seventy-seven patients were diagnosed to have grade IV GBM, 6 patients had grade III glioma with nonmutated IDH, and 15 patients were diagnosed by imaging only; therefore, their tumor grade and IDH1 status were indeterminate. Eighty patients were 75 years or older and the median age at the time of diagnosis was 73.3 years (65.2–97.4).

**Table 1.** Patient Characteristics

Covariate	Value	*N* (%)
Gender	Female	90 (45.5%)
	Male	108 (54.4%)
Histology	Grade IV	177 (89.4%)
	Grade III + nonmutated IDH	6 (3.0%)
	Indeterminate	15 (7.6%)
MGMT	Methylated	85 (42.9%)
	Unmethylated	89 (44.9%)
	Indeterminate	24 (12.1%)
EGFR amplification	Yes	52 (26.3%)
	No	123 (62.1%)
	Indeterminate	23 (11.6%)
Method of diagnosis	Resection	121 (61.1%)
	Biopsy	62 (31.3%)
	Radiological only	15 (7.6%)
R132H IDH1	Nonmutated	183 (92.4%)
	Indeterminate	15 (7.6%)
Age	65–70	53 (26.8%)
	70–75	65 (32.8%)
	>75	80 (40.4%)
Radiochemotherapy	Yes	116 (58.6%)
	Radiotherapy only	52 (26.3%)
	Cyclic TMZ only	3 (1.5%)
	No	27 (13.6%)
Cyclic temozolomide	Yes	80 (46.8%)
	No	91 (53.2%)
At least 6 cycles of TMZ	Yes	31 (38.8%)
	No	49 (61.3%)
Planned radiation scheme	59.4/1.8 Gy	13 (7.7%)
	60/2 Gy	31 (18.5%)
	40.05/2.67 Gy	30 (17.9%)
	30 or 39/3 Gy	88 (52.4%)
	20 or 25/4 or 5 Gy	6 (3.6%)
Number of treatment lines	0	15 (7.6%)
	1	126 (63.6%)
	2	45 (22.7%)
	3	12 (6.1%)
	Further relapse	6 (3.0%)
WHO performance status	0	20 (10.1%)
	1	49 (24.7%)
	2	69 (34.8%)
	3	40 (20.2%)
	4	14 (7.1%)
	Indeterminate	6 (3.0%)
Planned tumor volume	<200 cm^3^	62 (31.3%)
	200–400 cm^3^	78 (39.4%)
	>400 cm^3^	30 (15.2%)
	Indeterminate	28 (14.1%)

Abbreviation: EGFR, epidermal growth factor receptor; IDH1, isocitrate dehydrogenase 1; MGMT, O(6)-methylguanine-DNA-methyltransferase; TMZ, temozolomide.

After the diagnosis and possible surgery, 116 patients were assigned to radiochemotherapy, 52 patients received only radiotherapy, 3 patients received temozolomide cycles only, and 27 patients did not receive any further oncological treatments. The radiation therapy was initiated usually 2–4 weeks after the surgery. Most common radiation scheme was 30 or 39 Gy in fractions of 3 Gy^7^ and was scheduled for 88 patients (52.4%). The conventional course of 60 Gy in 30 fractions^1^ was planned for 31 patients (18.5%) and 40.05 Gy in 2.67 Gy fractions for 30 patients (17.9%). One hundred and forty-eight patients completed the scheduled radiation treatment while 20 patients did not. Thus, 88.1% of patients were able to complete their radiation treatment and 38.8% of patients were able to complete at least 6 cycles of the scheduled adjuvant temozolomide treatment.

On average, the patients received 1.3 different treatment lines with 63.6% of patients receiving 1 treatment round. Fifteen patients did not receive any oncological treatments; that is, their tumor was not resected, and they did not receive radiotherapy or cytotoxic drugs. Twelve patients were treated with third-line treatments, and 6 of them had a confirmed further relapse.

**Figure 1.**
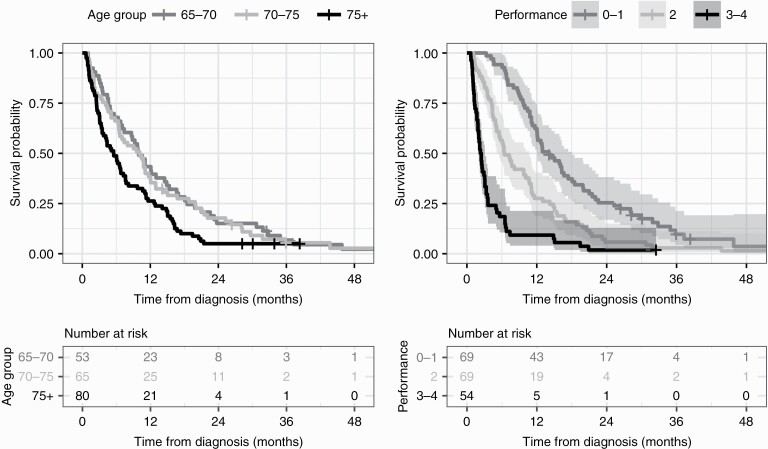
Kaplan–Meier survival curves by age group (left) and WHO performance status assessed at the onset of radiation therapy (right). The 95% confidence intervals are not drawn for age group-stratified survival curves for the sake of clarity. Below the survival curves are tables showing the number of patients alive at the beginning of each year in the different stratification groups.

OS and PFS are presented in Table 2 stratified to age groups. For patients 65–70 years old at the time of diagnosis, the median OS was 10.0 months (CI 7.7–12.3 months). For the age groups 70–75 years and >75 years the median OS were 10.1 months (CI 6.7–13.5 months) and 5.5 months (3.6–7.4 months), respectively. For the whole patient group, OS estimates at 6, 12, 18, and 24 months were 59.6% (53.1–66.8), 34.8% (28.8–42.2), 20.6% (15.6–27.0), and 11.8% (8.1–17.3), respectively. OS stratified by age groups is plotted in Figure 1.

**Table 2.** Kaplan–Meier Estimates for Overall and Progression-Free Survival Stratified by Age Groups

Age Group [years]	OS (95% CI) [months]	PFS (95% CI) [months]
65–70	9.95 (7.39–14.88)	6.93 (5.75–8.67)
70–75	10.12 (6.54–12.06)	6.74 (5.09–9.69)
>75	5.54 (3.75–7.62)	4.37 (3.52–6.21)

Abbreviations: OS, overall survival; PFS, progression-free survival.

To analyze the predictive value of various covariates known at the time of diagnosis on the OS, we modeled the data with the multivariable Cox regression model. A total of 59 patients were excluded due to missing either MGMT methylation, epidermal growth factor receptor amplification, WHO performance status, or planned tumor volume (PTV). Unmethylated MGMT, radiological diagnosis, and higher WHO performance status grade were statistically significantly related to shorter survival, see Table 3. In multivariate regression analysis in which WHO performance status was excluded (results not shown here), increasing age was statistically significantly correlated with a shorter survival. This implies that increasing WHO performance status and increasing age are correlated but WHO performance status is the one that correlates with shorter survival. Cross-tabulating performance status versus age groups and applying Pearson’s chi-squared test yields χ ^2^ = 12.22 and *P* = .016 for 4 degrees of freedom implying that covariates truly are not independent of each other. Especially, among patients over 75 years, there are more than expected patients with WHO performance status 3–4 and less than expected patients with WHO performance status 0–1.

**Table 3.** Multivariate Cox Regression Mode

Covariate	Value	Hazard Ratio	95% CI		*P* value
Age	Per 1-year increase	1.003	0.968	1.039	0.881
Gender	Male vs female	1.169	0.813	1.679	0.400
Underlying health condition	No vs yes	1.037	0.704	1.529	0.853
Multifocal	No vs yes	0.839	0.504	1.396	0.498
MGMT	Unmethylated vs methylated	1.726	1.188	2.508	4.2e-3
EGFR amplification	No vs yes	1.316	0.880	1.966	0.181
Diagnosis	Biopsy vs resection	2.223	1.398	3.535	7.4e-4
WHO perf. status	0–1 vs 2 vs 3–4	1.674	1.367	2.051	6.5e-7
PTV	Per 100-cm^3^ increase	1.061	0.987	1.141	0.107

Abbreviations: EGFR, epidermal growth factor receptor; MGMT, O(6)-methylguanine-DNA-methyltransferase; PTV, planned tumor volume. *N* = 139, number of events = 132.

To test the time-independency assumption of the Cox regression model hazards, the Grambsch and Therneau test of proportional hazards was applied.^9^ For the WHO performance status, the test gives χ ^2^ = 32.1 and *P* = 1.5e-8 implying time dependency. The scaled Schoenfeld residuals for the WHO performance status are plotted against the logarithmic time in Supplementary Material. The observed time dependency can be modeled fitting a linear relation in logarithmic time, β _WHO_(*t*) = 1.676 − 0.5459 log(*t*). The modified multivariate Cox regression model with time-dependent WHO performance status hazard coefficients is shown in Table 4. The time-dependent relative hazard per 1-grade increase in WHO performance status is 5.345 at 1 month and 2.965 at 1 year after the diagnosis. Keeping only the statistically significant covariates, for example, leaving out EGFR amplification, PTV, age, gender, underlying health conditions, and multifocality, decreased the number of excluded patients to 29. The multivariate Cox model on PFS gives similar results except for PTV which has hazard ratio 1.092 (95% CI 1.013–1.177) per 100-cm^3^ increase (*P* = .022).

**Table 4.** Modified Cox Regression Model With Time-Dependent WHO Performance Status Coefficient and Only Relevant Covariates

Covariate	Value	Hazard Ratio	95% CI		*P* value
MGMT	Unmethylated vs methylated	1.969	1.419	2.732	5.1e-5
Diagnosis	Biopsy vs resection	2.560	1.786	3.669	3.1e-7
WHO perf. status constant term	Per 1-grade increase	5.559	3.677	8.406	4.2e-16
WHO perf. status linear log(*t*) term	Per 1-grade increase	0.570	0.470	0.690	8.6e-9

Abbreviations: MGMT, O(6)-methylguanine-DNA-methyltransferase; *N* = 169, number of events = 161.

**Figure 2.**
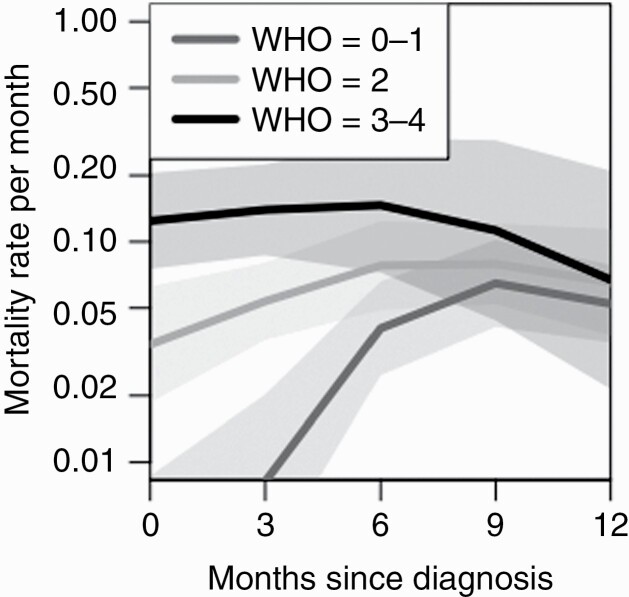
Mortality rates of different WHO performance groups in the Poisson model with time-dependent hazard for the groups. The mortality rates are for patients with methylated MGMT and resected tumor. The poorest performing patients have significantly higher risk during the first 6–9 months after which the better performing patients have risk increased to similar level.

The Cox proportional hazard model inherently assumes baseline hazard that is constant and equal for all groups. To gain further insight of the time dependency, we estimated the baseline hazards of different WHO performance groups using Poisson modeling.^10^ In Figure 2, mortality rates for these groups are presented for patients with methylated MGMT and resected tumor. In order to obtain a sufficient number of patients, the timescale spans 1 year. After 1 year the number of patients with original WHO performance score 3–4 is only 5 and confidence intervals become too large. The Poisson modeling reveals that patients with poor performance have higher risk for death already at the time of diagnosis. Patients with good performance have very low risk initially but the risk increases over time and becomes identical 6–9 months after the diagnosis.

## Discussion

### Key Findings

The aim of the present single-center retrospective real-life study was to study if the median OS of Finnish elderly patients with GBM has continued to increase since 2013. We found that during 2014–2018 the OS for GBM patients of 65–70 years, 70–75 years, and > 75 years old was 10.0 months, 10.1 months, and 5.5 months, respectively. Moreover, the median OS of those 17 patients aged over 75 years with WHO score 0–1 was as good as 14.1 months. Compared with the results of Korja et al., the median OS of the elderly has increased.

Our results imply that good performance status has the strongest correlation to and largest effect on longer OS. Also methylated MGMT and resection were correlated with longer survival. Interestingly, age was not independently correlated with longer survival although the patient group aged over 75 years had increased proportion of patients with WHO performance status 3–4 and decreased proportion with 0–1. Thus, our data suggest that age itself is not to be taken as a sign of poor prognosis.

Increasing PTV had a statistically significant effect on the PFS but not on the OS. It is possible that a very modest risk increase exists also on the OS, but our sample size was too small to detect it (relative risk 95% CI 0.983–1.132 per 100-cm^3^ increase in the time-dependent Cox model).

### Comparison With Previous Studies

Previously, in the study based on Finnish Cancer Registry’s material by Korja et al.,^8^ the median survival time in >70-year-old GBM patients increased from 3.6 months in 2000–2006 to 4.5 months in 2007–2013. Korja et al. had identified 2045 patients with GBM for their study involving 211 patients over 70 years during 2000–2006 and 308 during 2007–2013. Our study had quite a comparable number of 198 patients over 65 years diagnosed with GBM in a single Finnish center (CCC at HUH) during 2014–2018.

We presume that the main reasons for the differences regarding the OS results compared with the study of Korja et al.^8^ are the changes and development of the treatment practices since 2001–2006 and 2007–2013. Korja et al. reported that 13% of the elderly patients in 2005 and 33% of them in 2010 received chemoradiation compared to 58.6% of our patients during 2014–2018. While Korja et al. study was based on cancer register data, our data were collected from hospital records and this methodological difference could have also contributed to the OS discrepancy.

Before the era of Stupp’s protocol,^1^ our elderly patients had typically been treated with only radiotherapy if their condition allowed. The shortened schedule of radiation was preferred according to the study of Roa et al.^11^ In their prospective study containing 100 patients with GBM 60 years or over, it was found that hypofractionated radiotherapy (40/2.67 Gy) was not inferior to the 60/2 Gy treatment. OS times and the survival probabilities at 6 months were similar for standard and the shorter course of radiotherapy (5.1 vs 5.6 months and 44.7% vs 41.7%, respectively). After Stupp et al. published their study in 2005, chemoradiation plus adjuvant temozolomide became the new standard of care for GBM. However, even thereafter the 6-week long chemotherapy was not regularly applied for elderly patients (over 70 years) in our clinic since it was considered too heavy for the frail patients. During the period 2007–2013 elderly patients were treated according to their performance status in various ways.

Since 2012–2013 patients between 65 and 70 years were treated with Stupp’s protocol if their condition was adequate. For patients over 70 years hypofractionated (chemo)radiation 39/3 or 40.05/2.67 Gy was increasingly applied, succeeded by adjuvant temozolomide up to 6 cycles, if possible. In the present study, chemoradiation with a schedule of 30/3 or 39/3 Gy, with a schedule of 40.05/2.67 Gy, and with a schedule 59.4/1.8 or 60/2 Gy was performed for 52.4%, 27.9%, and 26.2% of the patients, respectively.

Furthermore, radiobiologically 40.05/2.67 Gy and 39/3 Gy are not so far from each other when counted as 2 Gy equivalent doses for normal brain tissue with alpha/beta value 2, 46.8 Gy versus 48.8 Gy. The 2 Gy equivalent tumor doses using tumor alpha/beta value 8 considered to be approximately the value for GBM tumors^12^ are close 42.7 Gy versus 42.9 Gy.

Comparing the OS result of our study with other previous studies of elderly GBM patients, quite similar results have been reported.^5–7^ In 2012, Wick et al.^6^ concluded in the NOA-08 study that for elderly patients with MGMT-methylated GBM, temozolomide alone is not inferior to 6-week long radiotherapy. In 2012 as well, in the Nordic trial Malmström et al.^5^ found that 6-week standard radiotherapy associates with poor outcome in GBM patients >70 years (median OS 6 months) and that temozolomide alone, especially in MGMT-methylated patients, or hypofractionated radiotherapy 34/3.4 Gy gave better results (median OS 8.3 and 7.5 months).

Perry et al.^7^ combined in their randomized trial hypofractionated radiotherapy 40.05/2.67 Gy with or without concurrent temozolomide followed by adjuvant temozolomide up to 12 cycles. There were 281 patients in each study group. They were 65 years or older and were considered unsuitable for the conventional treatment. The median OS was longer in the chemoradiation arm, 9.3 versus 7.6 months.

Nevertheless, our OS results fall clearly behind the results of a large-volume single-center study of Youssef et al.^13^ in which the median OS was 18.6 months for 158 GBM patients >65 years old for a period between 2001 and 2017. We suggest that the main reason for longer OS in their study is due to the better performance status. The authors state that patients referred to their center might have better performance status than in other centers. Our hospital receives practically all GBM patients of our responsibility area. Even the patients who are too ill to go through a histological biopsy pass via our clinic for an evaluation and discussion about the treatment possibilities. Thus, the patients with the poorest prognosis are included in our patient group. Almost 80% of the patients in the study of Youssef et al. (2019) had Karnofsky performance status (KPS) 80 or higher, whereas in our material only 35% had WHO classification performance status 0 or 1. Furthermore, over 90% of the patients got a 6-week course of postoperative chemoradiation followed by 6 cycles of adjuvant temozolomide. In comparison, 58.6% of the patients in our study received chemoradiotherapy. There were also differences in IDH and MGMT status discussed later.

The WHO performance status was most strongly correlated while age was not independently correlated with the OS in our study. This is in accordance with the results of a recent retrospective study of Al Feghali et al.^14^ Their study is based on the National Cancer Database Query between 2004 and 2015 of GBM patients with median age of 70 years at diagnosis. In the study the median OS was 10.45 months, 5.78 months, and 3.52 months in age groups 60–69 years, 70–79 years, and >80 years, respectively. Four groups based on age and KPS were created in the study. The study showed that the median survival was 4.96 months, 15.15 months, 9.59 months, and 6.83 months in group 1 (age > 60/KPS < 70), group 2 (60 to 69/KPS > 70), group 3 (age 70 to 79/KPS > 70), and group 4 (age > 80/KPS > 70), respectively. The investigators concluded that performance status is a key prognostic factor that should be considered when choosing between treatment options.

Regarding the length of chemotherapy, 38.8% of our patients were able to complete 6 cycles of the adjuvant temozolomide chemotherapy. Similarly, in the temozolomide-only arm of the Nordic study^5^ in which the intent was to complete 6 cycles, only 34% of the patients completed all 6 cycles and 86% reached at least 2 cycles. Youssef et al.^13^ reported that 38.6% of the patients completed the whole treatment including a 6-week course of chemoradiation with 6 cycles of temozolomide.

IDH and MGMT status were analyzed in as many as 183 (92%) and 174 (88%) of the GBMs in our study patients, respectively. One hundred and eighty-three patients who had a histological sample taken had also truly IDH1 wild-type gliomas. The amount of MGMT unmethylated patients was 45%. Unmethylated MGMT was statistically related to shorter survival. In the study of Youssef et al., the percentage of IDH1 wild-type gliomas was uncertain since 30.4% were not tested for IDH1 at all, and some IDH1 positive patients were included in the study as well. Moreover, MGMT status was not examined in the material of Youssef et al. in around 70% of patients, and the known unmethylated cases were only 11.4% of the whole material.

### Limitations and Strengths of the Study

Our study is a retrospective, single-center study including only a restricted number of elderly patients with GBM. All these 3 facts can be considered as limitations of the study. On the other hand, our patient material is unselected, which is an advantage of the study. All elderly patients with GBM of the District of Helsinki and Uusimaa in Finland were evaluated for therapy at our hospital, and not only those whose condition is good enough to receive radio- or chemotherapy. Furthermore, our treatment practices and principles of treatment were based on uniform standardized instructions of our hospital and were not dependent on any opinion of a single doctor.

## Conclusion

To conclude, this retrospective study shows that the prognosis of elderly GBM patients in our clinic has become better during past years with the accumulating knowledge about the treatment. The performance status of the individual patient is more important than age when choosing treatment protocols, which is in line with the results of other recent studies.

## Supplementary Material

npac008_suppl_Supplementary_Material

## References

[1] Stupp R, Mason WP, van den Ben J, et al. Radiotherapy plus concomitant and adjuvant temozolomide for glioblastoma. N Engl J Med. 2005;352(10):987–996. 15758009 10.1056/NEJMoa043330

[2] Paszat L, Laperriere N, Groome P, et al. A population-based study of glioblastoma multiforme. Int J Radiat Oncol Biol Phys. 2001;51(1):100–107. 11516858 10.1016/s0360-3016(01)01572-3

[3] Laperriere N, Weller M, Stupp R, et al. Optimal management of elderly patients with glioblastoma. Cancer Treat Rev. 2013;39(4):350–357. 22722053 10.1016/j.ctrv.2012.05.008

[4] Ostrom QT, Gittleman H, Liao P, et al. CBTRUS statistical report: primary brain and central nervous system tumors diagnosed in the united states in 2007–2011. Neuro Oncol. 2014;16(suppl_4):iv1–i63. 25304271 10.1093/neuonc/nou223 PMC4193675

[5] Malmström A, Grønberg BH, Marosi C, et al. Temozolomide versus standard 6-week radiotherapy versus hypofractionated radiotherapy in patients older than 60 years with glioblastoma: the Nordic randomised, phase 3 trial. Lancet Oncol. 2012;13(9):916–926. 22877848 10.1016/S1470-2045(12)70265-6

[6] Wick W, Platten M, Meisner C, et al. Temozolomide chemotherapy alone versus radiotherapy alone for malignant astrocytoma in the elderly: the NOA-08 randomised, phase 3 trial. Lancet Oncol. 2012;13(7):707–715. 22578793 10.1016/S1470-2045(12)70164-X

[7] Perry JR, Laperriere N, O’Callaghan CJ, et al. Short-course radiation plus temozolomide in elderly patients with glioblastoma. N Engl J Med. 2017;376(11):1027–1037. 28296618 10.1056/NEJMoa1611977

[8] Korja M, Raj R, Seppä K, et al. Glioblastoma survival is improving despite increasing incidence rates: a nationwide study between 2000 and 2013 in Finland. Neuro Oncol. 2018;21(3):370–379. 10.1093/neuonc/noy164 PMC6380416 30312433

[9] Grambsch PM, Therneau TM. Proportional hazards tests and diagnostics based on weighted residuals. Biometrika. 1994;81(3):515–526.

[10] Carstensen B . Who Needs the Cox Model Anyway? 2019. http://bendixcarstensen.com/WntCma.pdf. Accessed February 4, 2022.

[11] Roa W, Brasher PMA, Bauman G, et al. Abbreviated course of radiation therapy in older patients with glioblastoma multiforme: a prospective randomized clinical trial. J Clin Oncol. 2004;22(9):1583–1588. 15051755 10.1200/JCO.2004.06.082

[12] Pedicini P, Fiorentino A, Simeon V, et al. Clinical radiobiology of glioblastoma multiforme. Strahlenther Onkol. 2014;190(10):925–932. 24699988 10.1007/s00066-014-0638-9

[13] Youssef M, Ludmir E, Mandel J, et al. Hout-18. Treatment strategies for glioblastoma in older patients: age is just a number. Neuro Oncol. 2019;21(supplement_6):vi115–vi116. 10.1007/s11060-019-03304-x 31643011

[14] Al Feghali KA, Buszek SM, Elhalawani H, et al. Real-world evaluation of the impact of radiotherapy and chemotherapy in elderly patients with glioblastoma based on age and performance status. Neurooncol Pract. 2020;8(2):199–208. 33898053 10.1093/nop/npaa064 PMC8049423

