# Supplementary material for: Retrospective single-center study on elderly patients with glioblastoma between 2014 and 2018 evaluating the effect of age and performance status on survival
Source: Neurooncol Pract. 2022 Jan 27;9(2):142–8. doi: 10.1093/nop/npac008 (PMC8965048; doi:10.1093/nop/npac008)
Supplement: npac008_suppl_Supplementary_Material [file npac008_suppl_supplementary_material.docx]

# WHO performance status and treatment plan

The distribution of WHO performance status versus age is shown in Table 1. Pearson’s Chi-squared test gives χ^2^ = 12.21 (for 4 degrees of freedom p = 0.016). This implies that age groups and WHO performance status are correlated. Especially, among patients over 75 years there are fewer than expected patients with WHO performance status 0-1 and more than expected patients with WHO 3-4.

| WHO status | Age 65-70 | 70-75 | >75 | Total |
| --- | --- | --- | --- | --- |
| 0-1 | 23 | 29 | 17 | 69 |
| 1-2 | 16 | 22 | 31 | 69 |
| 3-4 | 13 | 12 | 29 | 54 |
| Total | 52 | 63 | 77 | 192 |

Table 1 WHO performance status versus age at the time of diagnosis.

To gain further insight to the ways patients were treated, chosen treatment protocol stratified by performance status was studied. The WHO performance status versus planned radiation scheme is tabulated in Table 2. None of the patients with the best performance rating was treated with palliative radiation schemes (20-25 Gy) and none of the patients with the worst rating underwent the heaviest radiation schemes (59.4/60 Gy).

| WHO status | Total dose of the radiation scheme | | | |
| --- | --- | --- | --- | --- |
|  | 59.4/60 Gy | 30-40.05 Gy | 20-25 Gy | Total |
| 0-1 | 31 (44.9 %) | 36 (52.2 %) | 0 (0 %) | 67 (97.1 %) |
| 2 | 12 (17.4 %) | 50 (72.5 %) | 1 (1.4 %) | 63 (91.3 %) |
| 3-4 | 0 (0 %) | 29 (53.7 %) | 4 (7.4 %) | 33 (61.1 %) |
| Total | 43 | 115 | 5 | 163 |

Table 2 WHO performance status and the total planned radiation dose. The percentage in the parentheses is the proportion of patients with the planned radiation scheme out of all patients with the same WHO performance rating.

The WHO performance status versus planned radiation scheme is tabulated in Table 3. Pearson’s Chi-squared test yields χ^2^ = 76.21 (p = 1.1e-15) which shows that the chosen radiation scheme was tightly correlated with the performance status.

| WHO status | Chemoradiation | Radiation only | No radiotherapy | Total |
| --- | --- | --- | --- | --- |
| 0-1 | 62 (89.9 %) | 5 (7.2 %) | 2 (2.9 %) | 69 (100 %) |
| 2 | 43 (62.3 %) | 20 (29.0 %) | 6 (8.7 %) | 69 (100 %) |
| 3-4 | 8 (14.8 %) | 25 (46.3 %) | 21 (38.9 %) | 54 (100 %) |
| Total | 113 | 50 | 29 | 192 |

Table 3 WHO performance status and the chosen radiation treatment scheme. The percentage in the parentheses is the proportion of patients with the planned radiation treatment out of all patients with the same WHO performance rating.

# Time-dependent effect of the WHO performance rating

The scaled Schoenfeld residuals for the WHO performance status are plotted against the logarithmic time in Figure 1. The observed time-dependency can be modeled fitting a linear relation in logarithmic time, β_WHO_(t)=1.676 – 0.5459 log(t).

Figure 1 - Scaled Schoenfeld residuals for WHO performance status as a function of logarithmic time. Black line denotes the fourth order and red line the second order polynomial fit to the data points. The dashed green line denotes the time-independent fit to the data points.
